# Direct comparison of circulating tumor DNA sequencing assays with targeted large gene panels

**DOI:** 10.1371/journal.pone.0266889

**Published:** 2022-04-28

**Authors:** Lizhi Yu, Gonzalo Lopez, John Rassa, Yixin Wang, Tara Basavanhally, Andrew Browne, Chang-Pin Huang, Lauren Dorsey, Jin Jen, Sarah Hersey

**Affiliations:** 1 Translational Sciences and Diagnostics, Translation Medicine, Bristol Myers Squibb, Summit, New Jersey, United States of America; 2 Translational Bioinformatics, Informatics and Predictive Sciences, Bristol Myers Squibb, Summit, New Jersey, United States of America; 3 Translational Research, Immuno-Oncology and Cell Therapy, Bristol Myers Squibb, Seattle, Washington, United States of America; University of California Irvine, UNITED STATES

## Abstract

Next generation sequencing (NGS) assays with large targeted gene panels can comprehensively profile cancer somatic mutations in a tumor sample. Given the rapid adoption of such assays for circulating tumor DNA (ctDNA) analysis in clinical oncology, it is essential for the community to understand their analytical performance in liquid biopsy settings. Here, we directly compared five ctDNA NGS assays, most of which having a panel of 400 or more genes, with simulated samples harboring mutations relevant to solid tumors or myeloid malignancy. Our results indicate that the detection sensitivity and reproducibility of all five assays was 90% or higher when the mutations were at 0.5% or 1.0% allele frequency, and with optimal DNA input of 30 ng or 50 ng per vendor’s protocol. The performances decreased and varied dramatically, when mutations were at a 0.1% allele frequency and/or when a lower genomic input of 10 ng DNA was used. Interestingly, one of the assays repeatedly showed higher rate of false positivity than the others across two different sample sets. Multiple intrinsic technical factors pertaining to the NGS assays were further investigated. Notable differences among the assays were seen for depth of coverage and background noise, which profoundly impacted assay performance. The results derived from this study are highly informative and provide a framework to assess and select suitable assays for specific application in cancer monitoring and potential clinical use.

## Introduction

Circulating tumor DNA (ctDNA) in plasma has been widely adopted as source of informative biomarkers for cancer early detection, patient stratification, efficacy monitoring, and post-treatment surveillance [1]. It is not only easily accessed by blood collection with minimal invasion, but also considered more homogenous and could reduce sampling bias within the tumor and across multiple focal disease sites [2].

Next-generation sequencing (NGS) is the most common method for ctDNA analysis, as it enables comprehensive profiling of many somatic cancer mutations within a single plasma sample [3, 4]. Advances in NGS technology and a tremendous demand for ctDNA analysis to support clinical studies have facilitated the emergence of sequencing assays covering several hundred cancer-related genes. By analyzing multiple genes for tumor-specific mutations, these large-panel NGS assays could be used for tumor mutational burden, ctDNA abundance assessments and to detect tumor specific mutations [5].

However, several inherent characteristics of ctDNA pose obstacles for NGS assays. Cell-free DNA exists as 160 to 200 base pair (bp) fragments [6] at low concentrations in plasma, which lead to challenges in acquiring enough quality material for sequencing, especially for large-panel NGS assays which usually require more DNA input to achieve high quality data. In addition, only a small fraction of cell-free DNA, often less than 1%, is ctDNA [7, 8]. The variant allele frequency (VAF) of tumor-derived mutations is often close to or below limit of detection of sequencing assays. Reliably detecting cancer-specific mutations with low VAF requires ctDNA assays with high sensitivity and specificity.

Various methods are used by different assay developers for cell-free DNA extraction, construction of NGS libraries, and target enrichment of informative cancer genes [9]. Downstream bioinformatics data analysis pipelines, including suppression of random errors from sequencing reactions and filtering out background mutations derived from artifacts of sequencing reactions [10] or germline DNA, also have the potential to strongly impact the results. It is critical for investigators to understand the sensitivity, specificity, accuracy and reproducibility of the assays, as well as the key technical factors that affect analytical performance, to select suitable sequencing assays for clinical studies. Several large-panel NGS assays are widely adopted by oncology community for ctDNA assessment, but reports are scarce regarding between-assay comparison of analytical performance or in-depth analysis of assay technical factors.

Here, we report the findings of a side-by-side evaluation of five leading ctDNA NGS assays. Four of the five assays have targeted panels of 400 or more genes, with the fifth assay featuring a flexible panel design that can cover over a hundred genes. Identical reference materials emulating clinical cell-free DNA samples were supplied to the assay vendors for direct comparability. Assay and vendor names were blinded to enable publication of data.

## Materials and methods

### Preparation of reference samples

Two sets of reference samples were prepared for the evaluation. Set one (Cat. # 0710–0140, 0141, 0143 and 0144, Seracare Life Sciences, Milford MA) comprised of a genomic DNA mixture extracted from diverse cancer cell lines and 40 reference mutations commonly occurred in solid tumors (S1 Table).

The sample set included four samples, carrying the reference mutations at VAF of 1%, 0.5%, 0.125%, and 0% (negative control), respectively. All the reference mutations in a sample were at the same VAF. DNA was fragmented to a size of 160–180 base pairs (bp) and was supplied in TE (10mM Tris-HCl pH7.5, 1mM EDTA) buffer at designated concentrations.

Sample set two was a custom designed panel (Seracare Life Sciences, Milford MA) also included four samples, each harboring 23 reference mutations related to myeloid malignancy (S2 Table) at 1%, 0.5%, 0.125%, and 0% VAF (negative control), respectively.

Sample set two contained multiple challenging to detect mutations (e.g. long insertions or deletions and a mutation near a tandem repeat region). In addition to fragmentation, DNA was spiked into synthetic plasma (Seracare Life Sciences, Milford MA) for sample set two to emulate clinical plasma samples.

VAF and fragment size of DNA in each sample in sample set one and two was verified per quality assurance procedures of the manufacturer.

### Assay performance evaluation

Five ctDNA assays (Table 1) were selected for the evaluation based on several criteria: 1) Ample published reports of assay utilization 2) Distinct technical features desirable for use in oncology clinical studies 3) Assays are developed and commercially available to support clinical research and 4) Willingness of assay vendors to participate in the study.

**Table 1:** Assay specifications from the vendors.

	Assay A	Assay B	Assay C	Assay D	Assay E
Size of Gene Panel	500	600	500	~500	~100
Mutation Type	SNV, Indel, CNA, Fusion	SNV, Indel, CNA/CNR, Fusion	SNV, Indel, CNA, Fusion	SNV, Fusion	SNV, Indel, CNA
Target Enrich	Hybrid Capture	Hybrid Capture	Hybrid Capture	Hybrid Capture	PCR
Input cfDNA	5-30ng	5-30ng	5-50ng	5-50ng	5-50ng

Abbreviation: SNV, single nucleotide variant; Indel, insertion/deletion; CNA, copy number amplification; CNR, copy number reduction; cfDNA, cell-free DNA

**Fig 1:**
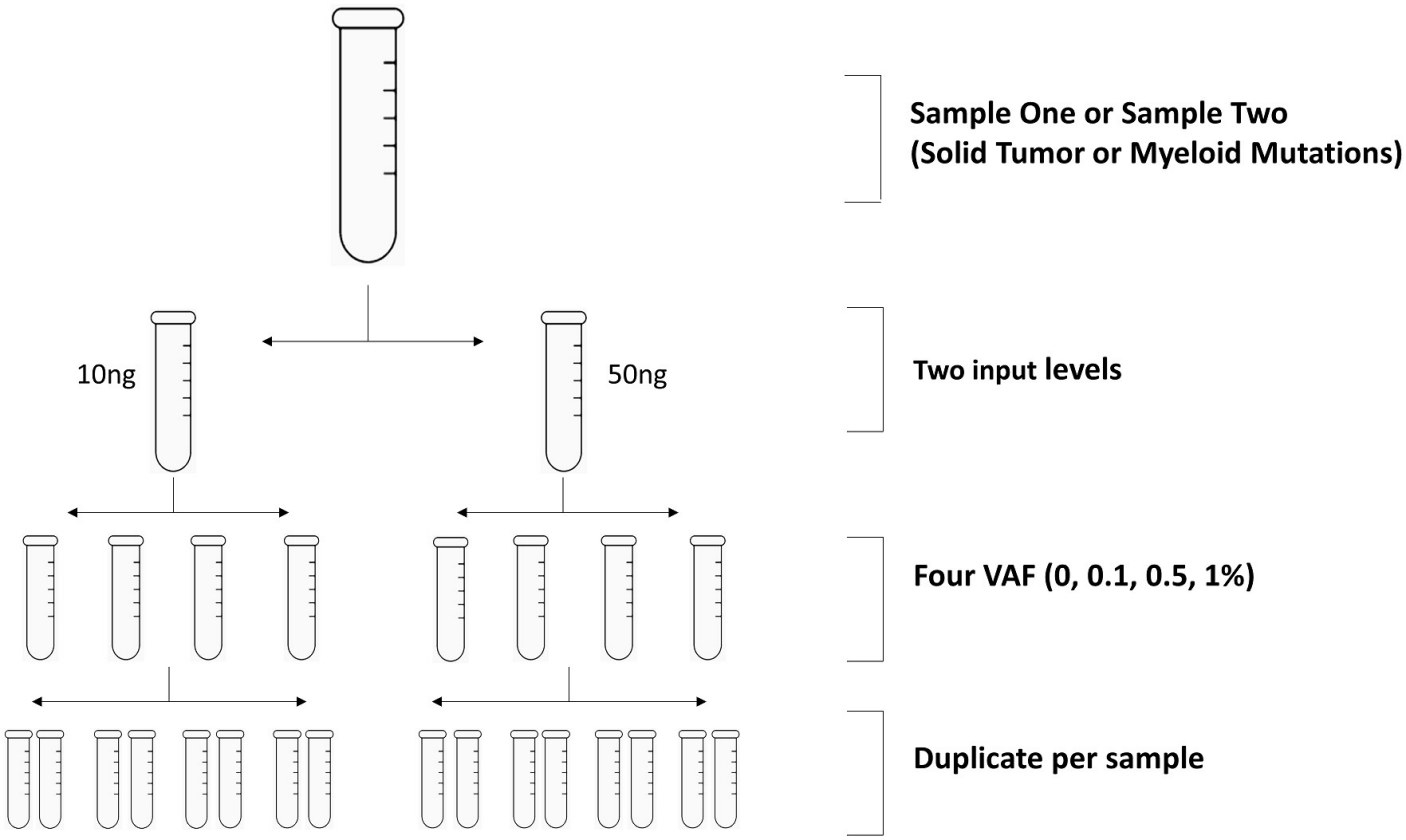
Schematic overview of the cross-platform study. Reference samples: Sample set one or set two tested at 30 ng or 50 ng (per vendor’s protocol specification, Table 1) and 10 ng input respectively. For each input level, four samples carrying mutations at VAF of 0%, 0.125%, 0.5% and 1% respectively were tested in duplicate.

Each assay vendor was supplied with aliquots of sample set one and set two, totaling to 16 samples per set. Each set includes samples with two DNA concentrations and four mutation VAF levels. Samples of each DNA concentration and VAF level are provided in duplicate vials for each vendor (Fig 1).

The sample and reference mutation information were blinded to the assay vendors, except for sample volume, DNA concentration, and sample matrix in each vial. Four assay vendors were supplied with both reference sample sets, since five or more of the reference mutations harbored in each sample set were covered by the assays (Table 2). The vendor of assay D was only supplied with sample set one due to a limited coverage of reference mutations in its gene panel for sample set two.

**Table 2:** Coverage of reference gene panel by assay A to E for reference samples.

**Sample Set One**	**Solid Tumor Mutation Panel**		**Assay A**	**Assay B**	**Assay C**	**Assay D**	**Assay E**
Mutation Type	Gene						
	n						
SNV	25	AKT1, APC, **BRAF**, CTNNB1, EGFR, FGFR3, **FLT3**, FOXL2, GNA11, GNAQ, GNAS, **IDH1**, **JAK2**, KIT, KRAS, **MPL**, NRAS, PDGFRA, PIK3CA, RET, SMAD4, TP53	25	25	25	5	24
Short Indel	13	APC, ATM, EGFR, ERBB2, **NPM1**, PDGFRA, PIK3CA, PTEN, SMAD4, TP53	12	13	13	0	13
Long Indel	0	N/A	0	0	0	0	0
Fusion	2	NCOA4-RET, TPR-ALK	2	2	2	0	1
**Sample Set Two**	**Myeloid Mutation Panel**		**Assay A**	**Assay B**	**Assay C**	**Assay D**	**Assay E**
Mutation Type	Gene						
	n						
SNV	14	ABL1, **BRAF**, CBL, CSF3R, **FLT3**, **IDH1**, **JAK2**, **MPL**, MYD88, SF3B1, U2AF1	13	14	14	2	14
Short Indel	4	*AXL1, CEBPA, JAK2, **NPM1**	3	3	3	1	3
Long Indel	5	AXL1, CALR, FLT3, SRSF2	5	5	5	0	5
Fusion	0	N/A	0	0	0	0	0

Boldface: reference mutations in both reference sample sets * the insertion is near a tandem repeat region

Each vendor tested the reference samples with their own laboratory procedures and bioinformatics data analysis pipelines. In addition to the final report, all vendors except vendor C provided additional data (e.g. unfiltered VCF, FASTQ or BAM files).

### Evaluation of results

Each vendor provided a comprehensive final report including mutations detected in each sample, depth of coverage, and number of reads to support each mutation call. To ensure direct comparability among the assays, a minimum of 4 reads of a variant allele was required for a mutation call. VAFs of the mutations detected were calculated through dividing the number of variant-supporting reads by the depth of coverage of the loci.

The analysis of assay performance was based on SNVs and short insert/deletions. Large insertion/deletions were not included in the analysis of assay performance due to poor detection for all the assays. Fusions/rearrangement were excluded from the analysis too due to the small number of this mutation type in the reference samples.

Detection sensitivity was defined as the number of reference mutations detected in at least one of the two replicates of each sample divided by the total number of the reference mutations covered by the assay.

False Positive Rate (FP) was defined as the number of reference mutations detected in at least one of the two replicates of negative control samples divided by the total number of the reference mutations covered by the assay.

Accuracy of observed VAF was defined as the consistency of the average of the reported VAF value versus the expected value of all the reference mutations. Reported VAF is 0% if a reference mutation is not detected.

Reproducibility was defined as the number of reference mutations detected in *both* replicates of each sample divided by the total number of the reference mutations covered by the assay.

Background noise was defined as the non-reference mutations detected after filtering out possible sequencing errors, artifacts and germline mutations through bioinformatics pipeline of the assays.

## Results

### Both VAF and DNA input affected detection sensitivity and reproducibility among the assays

**Fig 2:**
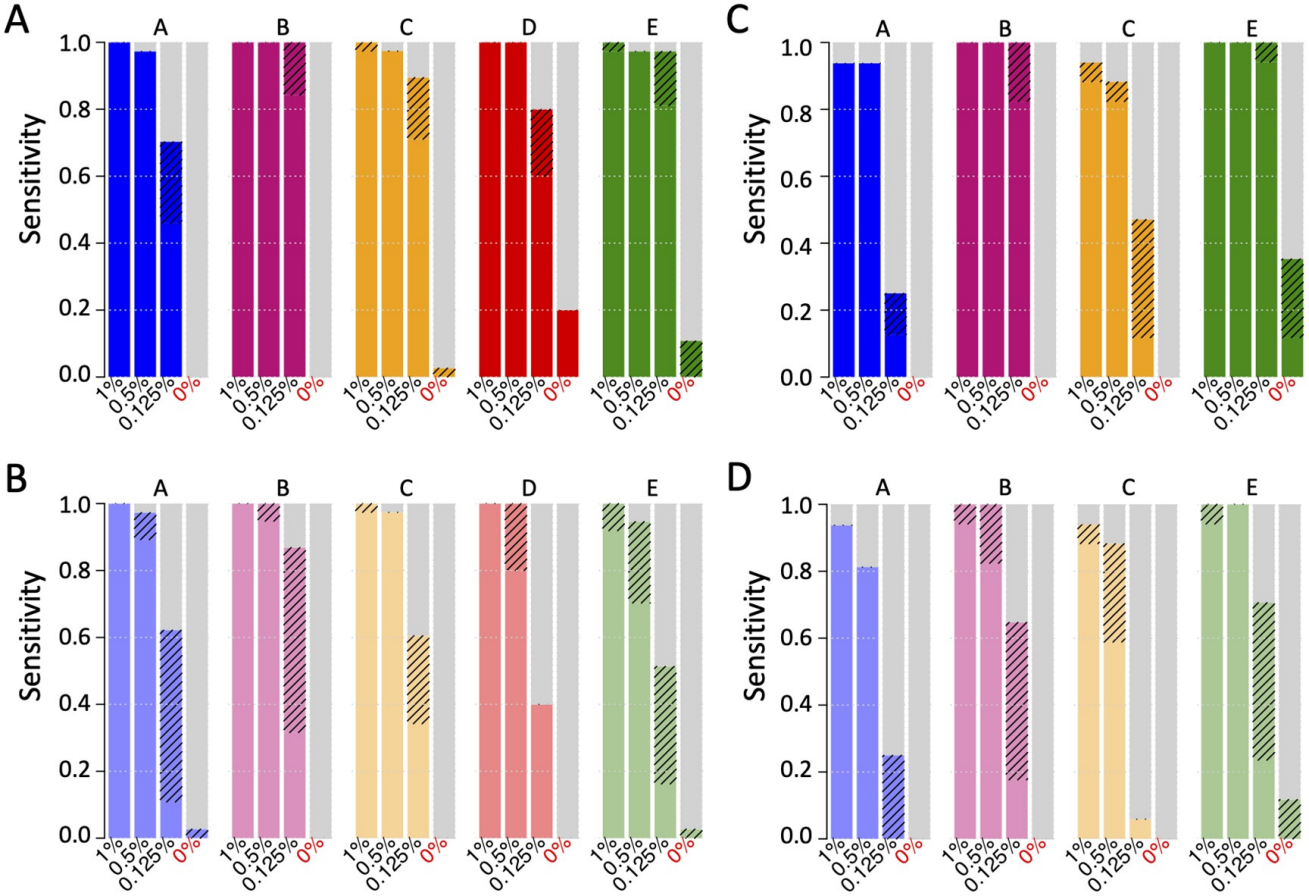
Analytical sensitivity and reproducibility. Solid bar: fraction of the reference mutations detected in both replicates; striped bar: fraction of the reference mutations detected in one of the two replicates; x-axis, VAF of the reference mutations; y-axis, detection sensitivity. A, Detection sensitivity (sum of solid and striped bar) and reproducibility (solid bar only) of each assay for sample set one at 0 to 1% VAF and input of 30 ng (assay A and B) or 50 ng (assay C, D, E). Assay B (purple) and assay E (green) detected 95–100% of the reference mutations at 0.125% VAF; B, Similar analysis for sample set one with 10 ng DNA input. Assay B (light purple) showed the highest sensitivity of 85%. C, Similar analysis for sample set two with input of 30 ng (assay A and B) or 50 ng (assay C, D, E). Assay B (purple) and assay E (green) exhibited 100% detection of the mutations at 0.125% VAF. D, Similar analysis for sample set two with 10ng DNA input. Assay B (light purple) achieved the highest detection sensitivity of 65% among the five assays.

For the mutations related to solid tumors (reference sample set one), all five assays demonstrated a high sensitivity (97–100%) when the VAF was at 0.5% or 1%, and DNA input was 30 ng or 50 ng (Fig 2A). The reproducibility was 100% at these VAFs and DNA input level for all the assays. At a VAF of 0.125%, the overall sensitivity and reproducibility decreased, but the impact on results differed among the assays. Assays B and E achieved >95% or higher sensitivity and a reproducibility of 80%, while the performance of the other three assays were poorer (sensitivity 70–90%, reproducibility 40–60%).

A lower DNA input also decreased the assay performance for solid tumor gene mutations, especially at the low VAF levels with varying impact across the assays. When DNA input was decreased to 10 ng, all five assays showed a high sensitivity (95–100%) at VAF of 0.5% or 1.0%, but only Assay A, B and C showed desirable reproducibility (90–98%, Fig 2B). At a VAF of 0.125%, the sensitivity was decreased to 90% for Assay B, and much lower for other assays (40–60%).

Similarly, VAF and DNA input levels also affected the detection of the myeloid cancer mutations in an assay-dependent mode. At 0.5% or 1.0% VAF, all assays showed a sensitivity of 90–100% and a reproducibility of 80–100% with 30 ng or 50 ng DNA (Fig 2C). At a 0.125% VAF, only Assays B and E achieved a sensitivity of 100% and reproducibility of 80–95% with 30 ng or 50 ng DNA, while Assays A and C showed sensitivity and reproducibility less than 50%. Similar to detection of the solid tumor mutations, the difference in assay performance was most dramatic when the VAF and DNA input were at the lower levels. With 10 ng DNA input, Assay B exhibited sensitivity of 65% at 0.125% VAF (Fig 2D). Assay E also showed similar sensitivity, however, the high false positive rate made the result questionable (see “**Analytical Specificity**” section below). The sensitivity of assay A and C was below 25%.

### Analytical specificity varied among the assays

We then evaluated analytical specificity in terms of false positive rate (FP). Because we used a pre-specified variant panel for a potential diagnostic assay, we defined FP as variants on the panel that are detected in negative samples (wild type samples). All five assays utilized unique molecular identifiers (UMIs) to suppress sequencing errors, and the FP of all four assays was below 2% (Table 3). Assay E showed a FP of 11% for the detection of solid tumor mutations and 35% for myeloid cancer mutations with a 50 ng DNA input, both of which were much higher than the other assays evaluated. With a 10 ng DNA input, Assay E also had a noticeable FP rate of 12% for the detection of myeloid cancer mutations. Although Assay D exhibited a FP of 20% for the detection of solid tumor mutations with a 50 ng input, the result should be considered preliminary since only a small number of mutations were covered by the assay.

**Table 3:** False positive rate.

**Solid tumor Mutations**		**Number of Mutations Detected**			**False Positive**
		**1%**	**0.50%**	**0.125%**	**0%**
**A**	**50ng**	37(100%)	36(97%)	26(70%)	0(0%)
	**10ng**	37(100%)	36(97%)	23(62%)	1(3%)
**B**	**50ng**	38(100%)	38(100%)	38(100%)	0(0%)
	**10ng**	38(100%)	38(100%)	33(87%)	0(0%)
**C**	**50ng**	38(100%)	37(97%)	34(89%)	1(3%)
	**10ng**	38(100%)	37(97%)	23(61%)	0(0%)
* **D**	**50ng**	5(100%)	5(100%)	4(80%)	1(20%)
	**10ng**	5(100%)	5(100%)	2(40%)	0(0%)
**E**	**50ng**	37(100%)	36(97%)	36(97%)	4(11%)
	**10ng**	37(100%)	35(95%)	19(51%)	1(3%)
**Myeloid Mutations**		**Number of Mutations Detected**			**False Positive**
		**1%**	**0.50%**	**0.13%**	**0%**
**A**	**50ng**	15(94%)	15(94%)	4(25%)	0(0%)
	**10ng**	15(94%)	13(81%)	4(25%)	0(0%)
**B**	**50ng**	17(100%)	17(100%)	17(100%)	0(0%)
	**10ng**	17(100%)	17(100%)	11(65%)	0(0%)
**C**	**50ng**	16(94%)	15(88%)	8(47%)	0(0%)
	**10ng**	16(94%)	15(88%)	1(6%)	0(0%)
**E**	**50ng**	17(100%)	17(100%)	17(100%)	6(35%)
	**10ng**	17(100%)	17(100%)	12(71%)	2(12%)

*5 reference mutations were covered by the assay

### The observed VAF for assays were consistent with the expected VAF in the samples

**Fig 3:**
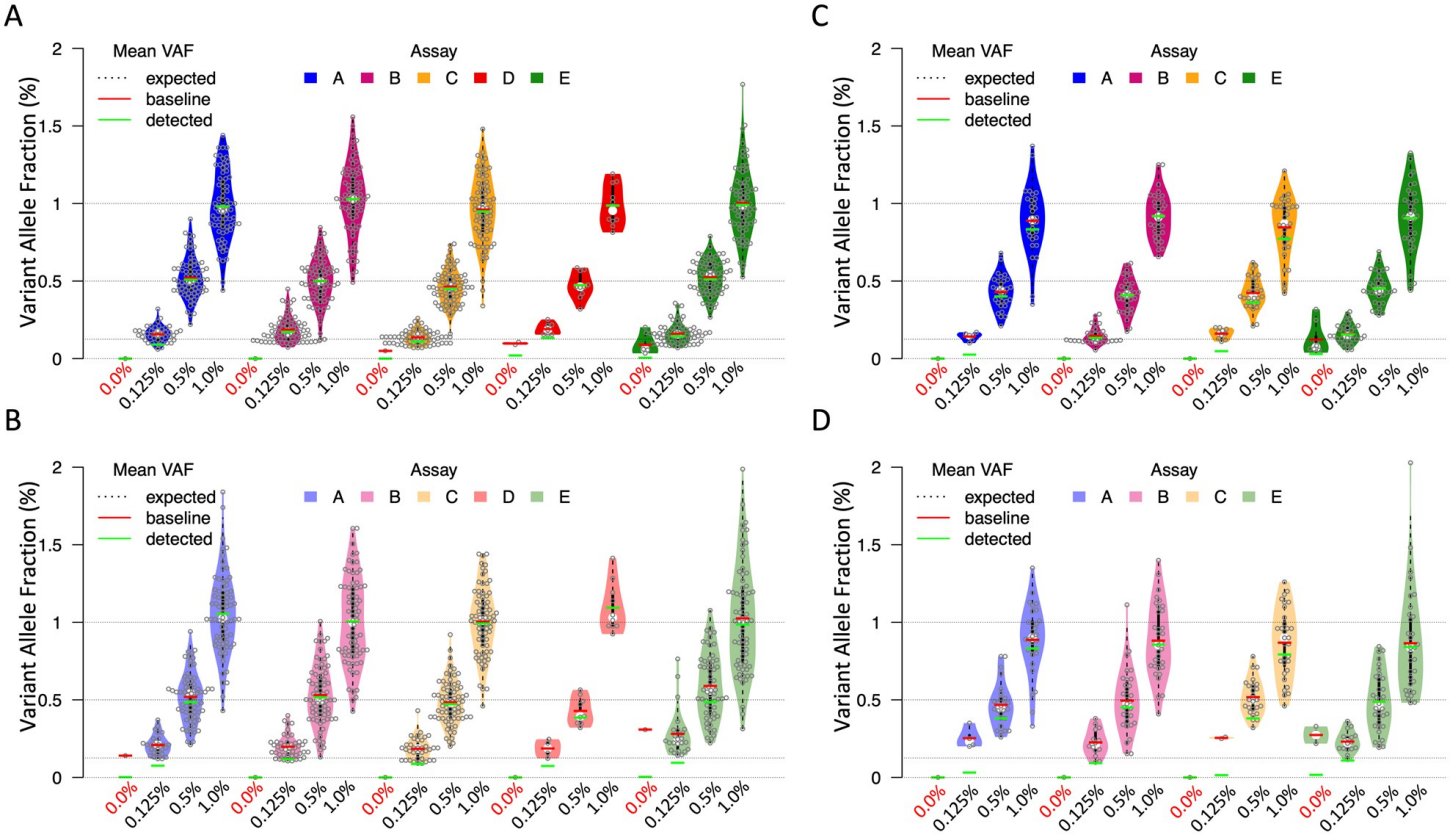
Quantification and accuracy of average VAF. Average VAF (green dash) was calculated using individual reported VAFs of all the reference mutations including 0% VAF values of non-detected mutations. The average VAF of only detected mutations (red dash) was also included for information purpose; the expected VAF is represented at 0%, 0.125%, 0.5% and 1% (dotted horizontal line). A, Distribution of the observed VAFs of the reference mutations in sample set one at 0 to 1% VAF and input of 30 ng (assay A and B) or 50 ng DNA (assay C, D, E); false positive mutations were detected by assay D (red) and assay E (green) from negative samples (violin plots at 0% VAF). B, Similar analysis for sample set one with 10 ng DNA input; average of observed VAFs was consistent with expected values for all five assays. False positive mutations were detected by assay A (light blue) and assay E (light green) from negative samples. C, Similar analysis for sample set two with 30 ng (assay A and B) or 50 ng (assay C, D, E) DNA input; average VAFs of assay B and assay E were closer to the expected values. Assay E (green) detected false positive mutations in negative samples. D, Similar analysis for sample set two with 10 ng DNA input; average VAFs of assay B and assay E were closer to the expected values. Assay E (green) detected false positive mutations in negative samples.

The observed VAF of solid tumor or myeloid cancer mutations were compared to the expected values for the samples. With a 30 ng or 50 ng and 10 ng DNA input, the average VAF of the reference mutations of solid tumors were close to the expected values (Fig 3A and 3B). For myeloid cancer mutations, the observed average VAF was slightly lower than the expected value of a 1% or 0.5% VAF level for all four assays. Assay B and assay E exhibited a more accurate average VAF than the other two assays for 0.125% VAF with a 30 ng or 50 ng DNA input (Fig 3C). With a 10 ng DNA input (Fig 3D), the observed VAF also slightly differed from the expected values. Assays B and E generated more accurate average VAF values than assay A and C, especially at 0.125% VAF level.

### The depth of coverage showed differences among the loci and the assays

**Fig 4:**
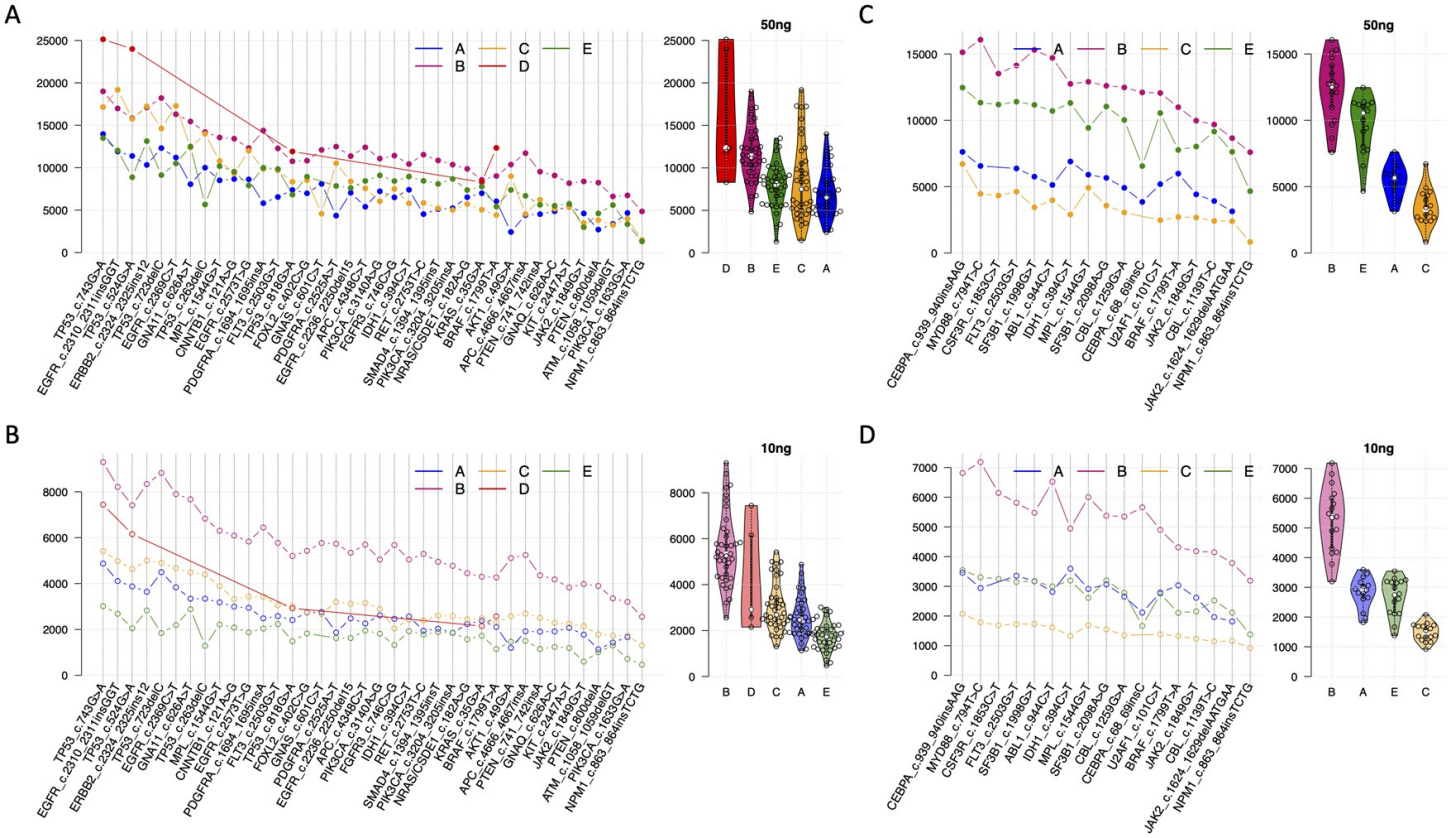
Depth of coverage and heterogeneity of the mutation loci. x-axis, mutation loci in sample set one or two; y-axis, depth of coverage after UMI consolidation. A, Depth of coverage of the mutation loci (line charts, left) and distribution (violin charts, right) for sample set one with 30 ng (assay A and B) or 50 ng (assay C, D, E). Assay B (pink) and assay D (red) showed higher depth of coverage than the other assays. B, Similar analysis for sample set one with 10 ng DNA input. Assay B has higher depth of coverage (light pink line, left) than the other assays across all the mutation loci and highest average coverage depth (light pink violin plot, right). C and D, Similar analysis for sample set two with 30 ng (assay A and B) or 50 ng (assay C, D, E) or 10 ng DNA input. assay B (dark or light pink lines, left) has higher depth of coverage than other assays across all the mutation loci and the highest average of coverage depth (dark or light pink violin plots, right).

Depth of coverage was evaluated for the gene mutations detected in the samples. For solid tumor mutations, while some loci had more than a 10,000-fold coverage, other loci only had a coverage of ~5,000-fold (Fig 4A, left). The difference of the coverage depth among loci was unrelated to the DNA input levels. The loci that had a high coverage with a 30 ng or 50 ng DNA input also showed a high coverage with a 10 ng DNA input, and vice versa for the loci that had a low coverage depth (Fig 4B, left). The between-loci difference was also observed for myeloid cancer mutations with a 30 ng or 50 ng (Fig 4C, left) and 10 ng DNA input (Fig 4D, left).

More importantly, the depth of coverage showed substantial differences among the five assays. For solid tumor mutations, Assays B and D exhibited the highest median coverage depth of ~ 12,000-fold with a 30 ng or 50 ng DNA input across 8 samples of various VAF levels (Fig 4A, right) compared to a coverage of 5,000 to 8,000-fold by the other three assays. The between-assay difference was more distinct with a 10 ng DNA input. Assay B achieved a median of a ~5,000-fold coverage depth, while the other assays merely reached a coverage depth of 1,000 to 3,000-fold (Fig 4B, right). Assay B also exhibited a consistently high coverage depth for myeloid cancer mutations: a ~12,000-fold with 30 ng DNA and a ~5,000-fold with 10 ng DNA, while the median coverage depth of other assays ranged from 2,000 to10,000-fold with 30ng or 50ng DNA (Fig 4C, right) and 1,000 to 3,000-fold with 10 ng DNA (Fig 4D, right).

It is also noticeable that the average depth of coverage for myeloid cancer mutations was lower than that of solid tumor mutations for all the assays at each of DNA input levels.

### Differentiated background noise were detected among the assays

**Fig 5:**
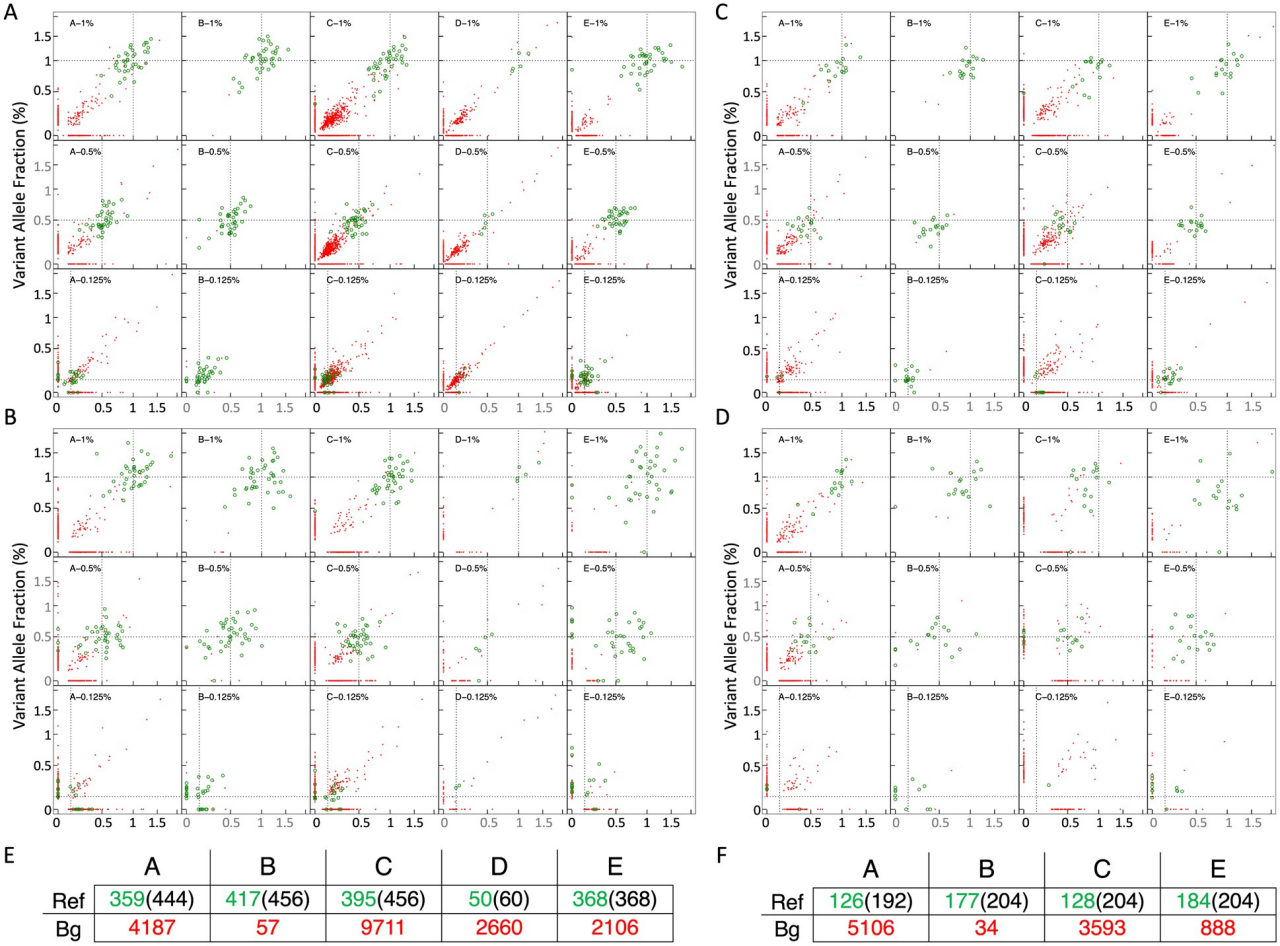
Reference mutations versus background noise across replicates. Every scatter plot displays a pair of replicates based on assay, VAF (0.125%, 0.5% and 1%) and input amount. Observed and expected VAF is represented in y and x axis; reference variants are colored in green and background noise in red. A, Background noise and VAF of reference mutations of solid tumor with 30 ng (assay A and B) or 50 ng (assay C, D, E). B, Same analysis for mutations of solid tumor with 10 ng input. C, Myeloid mutations with 30 ng (assay A and B) or 50 ng (assay C, D, E). D, Myeloid mutations with 10 ng. Through panel A to D, a high amount of background noise (red) was detected and overlapped with the lowest VAF level of the reference mutations evaluated (green) at 0.125% VAF for four of the five assays. Assay B showed exceptionally low background noise in all the samples. E, Summary table with number of detected reference mutations and background noise per assay for the solid tumor mutations (sum of 16 samples of sample set one); the total number of reference mutations covered by each assay was in parenthesis; ref, reference mutation; Bg, background noise. F, Similar summary table for the myeloid mutations (sum of 16 samples of sample set two).

Background noise was assessed through the number of non-reference mutations detected by the assays. We defined the background noise as variants detected in sequences that are not on the pre-specified panel. They could be due to sequencing errors insufficient filtered by the bioinformatics data analysis pipeline for each of the evaluated assays. For four of the five assays evaluated, thousands of background variants were detected across the 16 reference samples of sample set one, most of which showed a VAF of 0.1% (Fig 5A and 5B), which overlapped with the lowest VAF level of the reference mutations evaluated. A similar level of background noise was seen in sequencing results of the four assays evaluated for sample set two (Fig 5C and 5D). In comparison, Assay B filtered out 95% of the background noise and only detected 57 non-reference mutations in sample set one, and 34 in sample set two.

### Cell-free DNA extraction process did not impact assay performance

**Fig 6:**
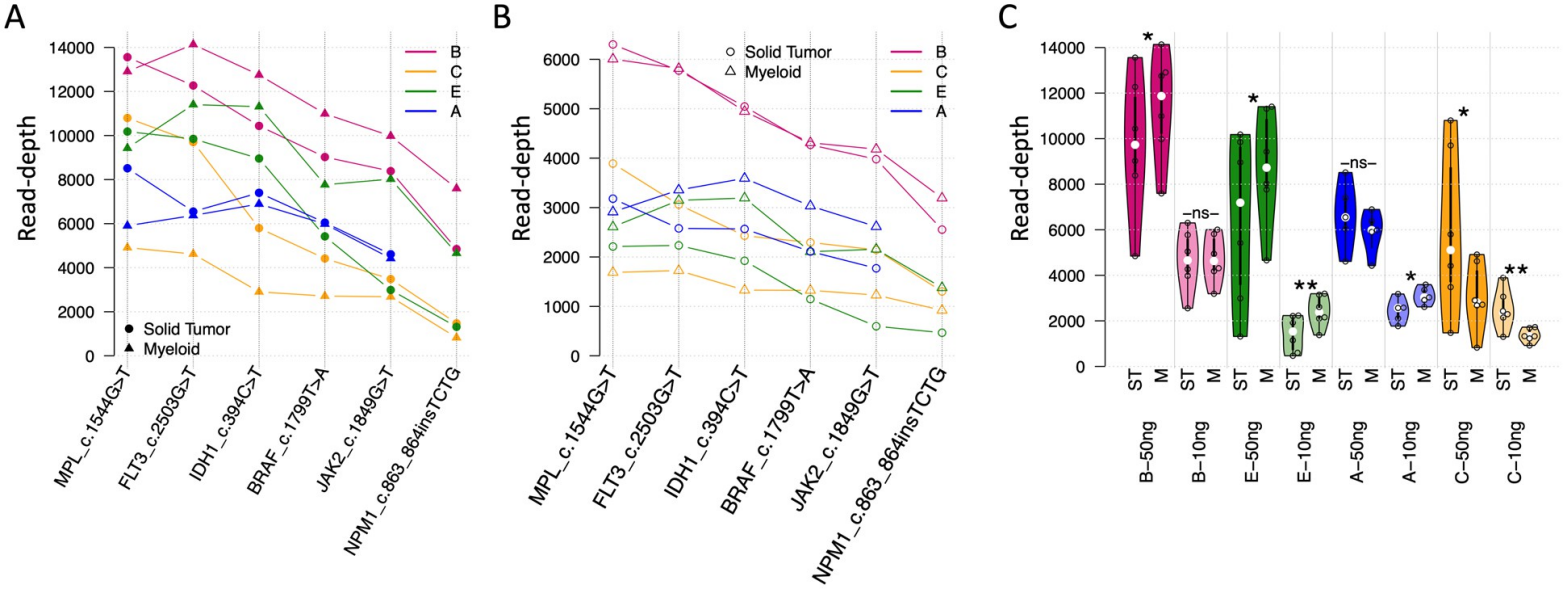
Impact of DNA extraction process on depth of coverage. Solid tumor or ST, reference samples carrying solid tumor mutations that were tested without DNA extraction; myeloid or M, reference samples carrying myeloid mutations that were tested including DNA extraction. A, Coverage depth for the 6 common mutations across the two reference sample sets for 30 ng (assay A and B) or 50 ng (assay C, D, E) DNA input. B, similar analysis for 10 ng input. C, Intra-assay comparison of coverage depth with vs. without DNA extraction process; statistical significance for the distribution mean differences measured by paired t-test (-ns- not significant, * <0.05, **<0.01). Overall, assay C (dark and light yellow violin plots) was the only one that tended to perform worse when DNA extraction was included.

Six common reference mutations were carried in both sample sets, which allowed for an evaluation of the impact of DNA extraction on the sequencing results; Sample set one was provided in TE solution that could be used for direct sequencing, while sample set two was in synthetic plasma which required DNA extraction prior to sequencing. With a 30 ng or 50 ng DNA input, the coverage depth of the six mutations were relatively consistent whether DNA extraction was conducted or not except for assay C (Fig 6). A similar pattern was observed with a 10ng DNA input.

### VAF was not impacted by mutation type and challenging sequence context affected mutations detection

The difference of observed vs. expected VAF of all the reference mutations was evaluated based on the mutation types. The result indicated no impact of mutation types (SNV, small insertion, small deletion) on the VAF accuracy (S1 Fig).

In addition, sample set two harbored six mutations that are challenging to detect, including five large insertions or deletions of more than 20 bp and one small insertion near a tandem repeated region (Table 4). Only one of the six mutations was detected by all four assays evaluated. The other 5 mutations were missed by some, if not all, of the assays, even at a 1% VAF and an optimal DNA input.

**Table 4:** Detection of the Challenging mutations at 1% VAF and 50 ng DNA input.

Gene ID	Mutation Loci	Detected
ASXL1	c.1900_1922del23	All four assays
ASXL1	c.1934_1935insG	None
CALR	c.1092_1143del52	None
FLT3	c.1759_1800dup	None
FLT3	Duplication of chr13:28,608,250–28,608,277	Assay C
SRSF2	c.284_307del24	Assay A and E

## Discussion

ctDNA sequencing is becoming rapidly adopted in translational medicine and clinical oncology. Since NGS assays with large gene panels provide a broader coverage of cancer-derived mutations, they are gaining popularity in research and development of anti-cancer therapeutics. Given the challenges associated with ctDNA and difficulties in acquiring high quality data with large gene panels in NGS assay [11], it is critical to understand the analytical performance of ctDNA sequencing assays in large-panel settings. To this end, a direct comparison of five selected NGS assays, most of which having a panel of more than 400 genes, was performed using contrived samples carrying mutations relevant to solid tumors or myeloid cancers.

The result indicated that all five assays were able to achieve a high sensitivity for mutations at a 0.5% VAF or higher with an optimal input DNA amount of 30 or 50 ng. The observed sensitivity at these VAF levels were similar to the results from the assay vendors (Table 1). However, only two assays, Assays B and E, maintained a high sensitivity when VAF of the mutations was 0.125%. The other assays exhibited a sensitivity below 90% even with 30 ng or 50 ng DNA input. The performance of the assays also differed significantly when the DNA input level was at 10 ng. Assay B was the only assay that achieved a near 80% sensitivity with 10 ng DNA input when detecting mutations at a 0.125% VAF. Reproducibility followed the same trend as sensitivity. The sequencing results were more reproducible for mutations at VAF level of 0.5% or higher, or when DNA input amount was 30 or 50 ng. Reproducibility of the assays decreased when the mutations were at low VAF levels, or when the DNA amount was low. A clear understanding of sensitivity and reproducibility of a given ctDNA assay is key to decide its suitability for use in clinical oncology. The assays with a higher sensitivity and reproducibility are more advantageous for molecular characterization of early-stage cancer or monitoring of minimal residual disease (MRD) for on-treatment or post-treatment patients due to the lower allele frequency of somatic mutations and scarcity of ctDNA in the samples. Development of more sensitive assays for MRD purposes is underway at some of the providers of the assays studied in this report.

It is important to note that one of the five assays, assay E, exhibited a FP of 6% or higher vs. a FP of 3% or lower in other assays, although UMI was used for error-suppression in all the assays included in our study. It did not seem to occur randomly since higher FP of assay E was observed in two separate studies using different sample sets. Interestingly, this assay is the only one that uses extensive PCR amplification for target enrichment, while the other assays use hybrid capture. PCR amplification allows for a flexible panel design which is required for certain applications, such as tumor-guided and personalized MRD analysis. It also helps to increase assay sensitivity, especially when DNA input is low [12]. However, some known shortcomings of PCR amplification e.g. higher risk of cross contaminating samples, might contribute to the observed high false positive rate. More extensive follow-up investigation with the assay provider is underway to gain insight on the results.

The dynamic change of ctDNA abundance at baseline and during treatment is an important indicator for monitoring early response to therapy or early disease relapse [13]. Since abundance of ctDNA is usually estimated based on average allele frequencies of cancer-derived mutations, it is important that ctDNA assays can accurately quantify VAF of somatic mutations. Our results indicated that the allele frequency of reference mutations of solid tumor measured by the assays is close to the expected value down to 0.125% VAF, but only when the DNA input is 30 ng or 50ng. With a low DNA input of 10 ng, measurements of VAF at 0.125% tend to be unreliable. For the reference mutations of myeloid cancer, the observed VAF was slightly lower than the expected values at 1% and 0.5% VAF level for all four assays evaluated. Interestingly, the average depth of coverage for myeloid mutations was generally lower than that of the solid tumor mutations for all the assays too. The lower depth of coverage might account for the higher level of inaccuracy of VAF and lower sensitivity as discussed below. This highlights the need of more sensitive assays capable of accurately assessing changes of tumor burden during patient treatment when the total cell-free DNA amount is low and the allele frequencies of cancer-derived mutations decrease. The use of algorithms that incorporate the dependence of VAF precision and position-specific coverage of mutations could potentially improve measurements of cfDNA based tumor burden and drive applications in therapy response monitoring.

Additional variables pertaining to NGS assays were analyzed. The depth of coverage for mutation loci differs significantly among the assays. For example, Assay B consistently demonstrated a higher coverage depth than the other assays across mutations of different disease types, independent of VAF levels and DNA inputs. A higher coverage enables a more reliable detection of rare ctDNA mutations. This might partially account for the superior sensitivity of Assay B demonstrated across the two reference sample sets, especially for mutations at 0.125% VAF or when DNA input was low. The observed impact of coverage depth on assay performance is consistent with the results from other studies. Deveson et al. [14] used a set of sequenins of 0.1–100% VAF for a similar study and observed that a decreasing coverage had a strong negative effect on the detection of low-frequency mutations (VAF < 0.5%), whereas mutations at intermediate (0.5–5%) and high (>5%) frequencies were detected with a high sensitivity, even at low fragment depths. It should be noted that coverage depth is a collective outcome of multiple assay-intrinsic technical factors, including library construction, target enrichment, and PCR amplification. When given a standardized DNA input for each assay, the higher coverage depth of Assay B reflects its capacity to exhaustively recruit the unique DNA molecules in a sample into sequencing reactions. Compared to NGS assay with fixed and large gene panels, custom designed assays through tumor-guided approach often cover a much smaller number of genes. That potentially enables such assays to achieve higher depth of coverage with lower amount of DNA input and cost. However, all the underlying factors mentioned above regarding depth of coverage need to be carefully optimized to materialize the potential.

Bioinformatics data analysis pipeline is another critical step for error-suppression and filtering out background noise [15]. It is vital to achieve a high detection specificity since many background mutations are at similar VAF levels of true somatic mutations, which was observed in our study. The background noise may cause false positive results. However, over-stringent filtering might eliminate true somatic mutations from reporting and incur false negative read-outs. While most of the participating assays reported thousands of background mutations (non-reference mutations in our study) from the reference samples, the number of background mutations from Assay B is significantly lower than those from the others. In other words, Assay B eliminated over 95% of background noise through its bioinformatics pipeline without using a germline DNA sample as a control. This is impressive, considering Assay B also achieved the highest sensitivity among the five assays.

Because the coverage depth is an important technical factor regarding assay performance, it was measured for six reference mutations that were harbored in both the reference sample sets to evaluate the impact of DNA extraction. Except for Assay C, there is no significant negative impact of DNA extraction on coverage depth across the high and low DNA input levels. This analysis is preliminary due to the small number of mutations included.

Six challenging mutations including insertion > 20bp (2), deletion >20bp (3) and a small insertion near a tandem repeat region were intentionally included in the reference sample set two. Five of the six challenging mutations were missed by some, if not all, of the assays at all VAF and input levels evaluated, indicating the difficulties of detecting such mutations by NGS assays. On the contrary, assay performance on single nucleotide variants and small insertions or deletions did not significantly differ. Although the analysis is preliminary in nature, the observation is consistent with the outcomes from other studies [14, 16]. The assay performance for fusion/re-arrangement variants is not fully evaluated in the study due to 1) only a small number of such variants are carried in the reference samples. 2) most of genes breaking points involved in fusion/re-arrangement are unpredictable. So NGS assays with fixed gene panels, including the five assays evaluated in this study, usually cover a limited number of fusion/re-arrangement loci. Sequencing circulating tumor RNA in parallel can help to improve detection of fusion/re-arrangement variants.

The current study provides insight into the analytical characteristics of large gene panel ctDNA sequencing assays. These assays emerged in an increasingly number of research reports but were rarely assessed through direct comparison studies. Moreover, intrinsic technical factors pertaining to sequencing assays and their impact on analytical performances were investigated. The information derived from the study is crucial towards the ultimate goal of selecting the most suitable and reliable ctDNA assays for specific applications in clinical oncology.

## Supporting information

S1 Fig Impact of mutation type on VAF quantification performance. A, For each reference mutation of solid tumor, a distribution of the log-ratios of observed versus expected VAF from all VAF levels and input amounts is represented per assay. B, Similar analysis for reference mutations of myeloid cancer. Overall, the accuracy of VAF quantification was not associated the mutation types evaluated.
(TIF)

S1 Table Reference mutations of solid tumors. (DOCX)

S2 Table Reference mutations of myeloid malignancy. (DOCX)
