# Supplementary figures and images for: Direct comparison of circulating tumor DNA sequencing assays with targeted large gene panels
Source: PLoS One. 2022 Apr 28;17(4):e0266889. doi: 10.1371/journal.pone.0266889 (PMC9049497; doi:10.1371/journal.pone.0266889)

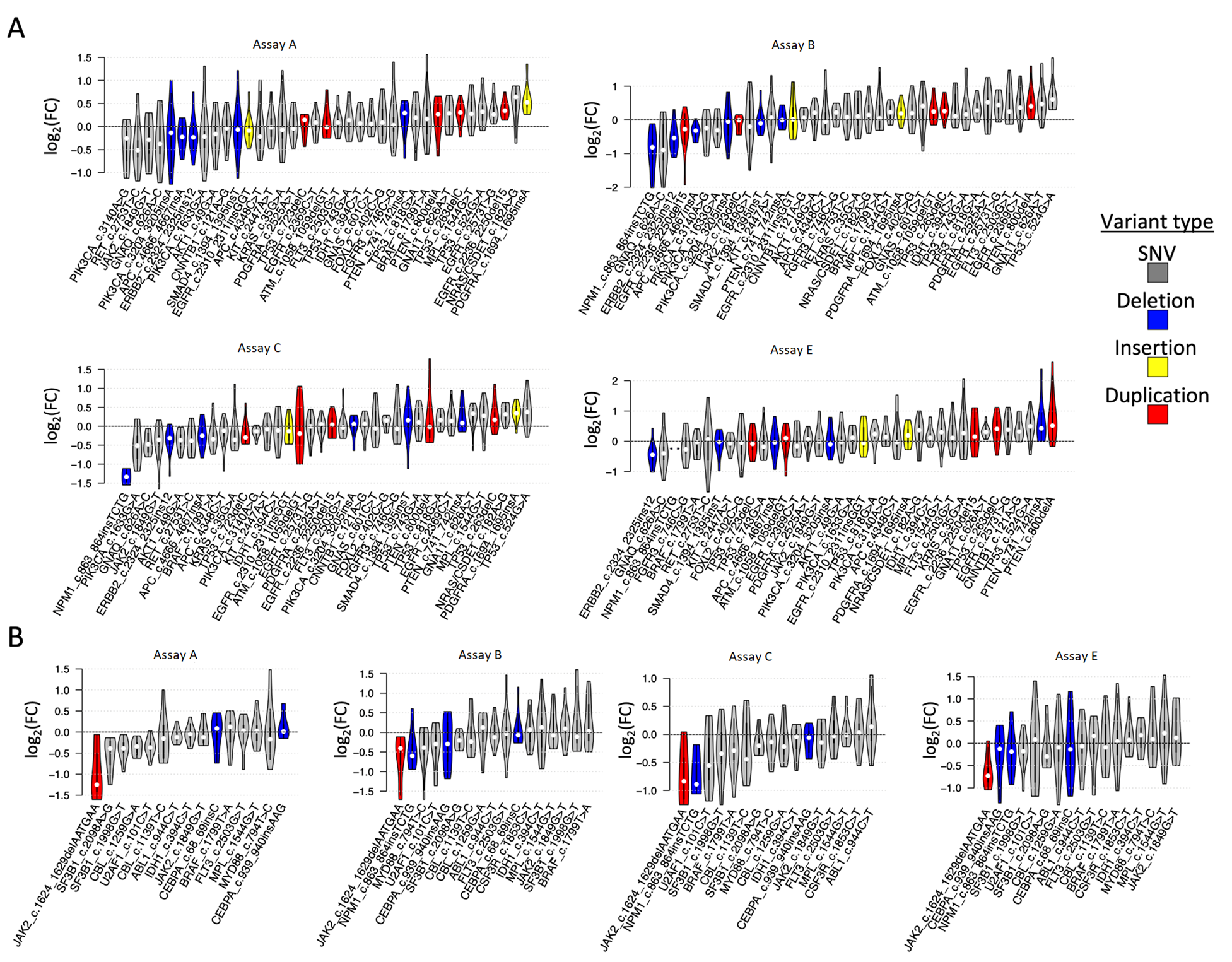

Supplement: S1 Fig — A, For each reference mutation of solid tumor, a distribution of the log-ratios of observed versus expected VAF from all VAF levels and input amounts is represented per assay. B, Similar analysis for reference mutations of myeloid cancer. Overall, the accuracy of VAF quantification was not associated the mutation types evaluated. (TIF) [file pone.0266889.s001.tif]
