# Supplementary material for: Direct comparison of circulating tumor DNA sequencing assays with targeted large gene panels
Source: PLoS One. 2022 Apr 28;17(4):e0266889. doi: 10.1371/journal.pone.0266889 (PMC9049497; doi:10.1371/journal.pone.0266889)
Supplement: S1 Table — (DOCX) [file pone.0266889.s002.docx]

S1 Table. Reference mutations of solid tumors

| **Gene ID** | **COSMIC ID** | **Mutation Type** | **HGVS Nomenclature** |
| --- | --- | --- | --- |
| AKT1 | COSM33765 | SNV | c.49G>A |
| APC | COSM13127 | SNV | c.4348C>T |
| APC | COSM18561 | Insertion | c.4666_4667insA |
| ATM | COSM21924 | Deletion | c.1058_1059delGT |
| BRAF | COSM476 | SNV | c.1799T>A |
| CTNNB1 | COSM5664 | SNV | c.121A>G |
| EGFR | COSM6224 | SNV | c.2573T>G |
| EGFR | COSM12378 | Insertion | c.2310_2311insGGT |
| EGFR | COSM6225 | Deletion | c.2236_2250del15 |
| EGFR | COSM6240 | SNV | c.2369C>T |
| ERBB2 | COSM682/20959 | Insertion | c.2324_2325ins12 |
| FGFR3 | COSM715 | SNV | c.746C>G |
| FLT3 | COSM783 | SNV | c.2503G>T |
| FOXL2 | COSM33661 | SNV | c.402C>G |
| GNA11 | COSM52969 | SNV | c.626A>T |
| GNAQ | COSM28758 | SNV | c.626A>C |
| IDH1 | COSM28747 | SNV | c.394C>T |
| JAK2 | COSM12600 | SNV | c.1849G>T |
| KIT | COSM1314 | SNV | c.2447A>T |
| KRAS | COSM521 | SNV | c.35G>A |
| MPL | COSM18918 | SNV | c.1544G>T |
| NCOA4-RET | N/A | Fusion | NCOA4{NC_000010.10}: r.1_1014+1312 RET{NC _000010.10}:r.2327-1437_5659 |
| NPM1 | COSM17559 | Insertion | c.863_864insTCTG |
| NRAS/CSDE1 | COSM584 | SNV | c.182A>G |
| PDGFRA | COSM736 | SNV | c.2525A>T |
| PDGFRA | COSM28053 | Insertion | c.1694_1695insA |
| PIK3CA | COSM763 | SNV | c.1633G>A |
| PIK3CA | COSM12464 | Insertion | c.3204_3205insA |
| PIK3CA | COSM775 | SNV | c.3140A>G |
| PTEN | COSM4986 | Insertion | c.741_742insA |
| PTEN | COSM5809 | Deletion | c.800delA |
| SMAD4 | COSM14105 | Insertion | c.1394_1395insT |
| TP53 | COSM10648 | SNV | c.524G>A |
| TP53 | COSM10660 | SNV | c.818G>A |
| TP53 | COSM10662 | SNV | c.743G>A |
| TP53 | COSM6530 | Deletion | c.723delC p.C242fs*5 |
| TP53 | COSM18610 | Deletion | c.263delC |
| TPR-ALK | N/A | Fusion | TPR{NC_000001.10}: r.1_2185+246_ALK{NC_000 002.11}:r.4125-550_6265 |

Abbreviation: SNV, single nucleotide variant
