# Supplementary material for: Direct comparison of circulating tumor DNA sequencing assays with targeted large gene panels
Source: PLoS One. 2022 Apr 28;17(4):e0266889. doi: 10.1371/journal.pone.0266889 (PMC9049497; doi:10.1371/journal.pone.0266889)
Supplement: S2 Table — (DOCX) [file pone.0266889.s003.docx]

S2 Table. Reference mutations of myeloid malignancy

| **Gene ID** | **COSMIC ID** | **Mutation Type** | **HGVS Nomenclature** |
| --- | --- | --- | --- |
| ABL1 | COSM12560 | SNV | c.944C>T |
| ASXL1 | COSM36165 | Deletion | c.1900_1922del23 |
| ASXL1 | COSM34210 | Insertion | c.1934_1935insG |
| BRAF | COSM476 | SNV | c.1799T>A |
| CALR | COSM1738055 | Deletion | c.1092_1143del52 |
| CBL | COSM34077 | SNV | c.1259G>A |
| CBL | COSM34055 | SNV | c.1139T>C |
| CEBPA | COSM18922 | Insertion | c.68_69insC |
| CEBPA | COSM18099 | Insertion | c.939_940insAAG |
| CSF3R | COSM1737962 | SNV | c.1853C>T |
| FLT3 | N/A | Insertion | c.1759_1800dup |
| FLT3 | N/A | Insertion | Duplication of chr13:28,608,250-28,608,277 (hg19), insGCCCC between duplicated and native Seq |
| FLT3 | COSM783 | SNV | c.2503G>T |
| IDH1 | COSM28747 | SNV | c.394C>T |
| JAK2 | COSM12600 | SNV | c.1849G>T |
| JAK2 | COSM24440 | Deletion | c.1624_1629delAATGAA |
| MPL | COSM18918 | SNV | c.1544G>T |
| MYD88 | COSM85940 | SNV | c.794T>C |
| NPM1 | COSM17559 | Insertion | c.863_864insTCTG |
| SF3B1 | COSM84677 | SNV | c.2098A>G |
| SF3B1 | COSM131557 | SNV | c.1998G>T |
| SRSF2 | COSM146289 | Deletion | c.284_307del24 |
| U2AF1 | COSM166866 | SNV | c.101C>T |

Abbreviation: SNV, single nucleotide variant
